# Measurement of Quality of Life I. A Methodological Framework

**DOI:** 10.1100/tsw.2003.75

**Published:** 2003-10-13

**Authors:** Soren Ventegodt, Jorgen Hilden, Joav Merrick

**Affiliations:** The Quality of Life Research Center, Copenhagen K, Denmark

**Keywords:** Quality of Life, QOL, measurement, screening, questionnaires, SEQOL, human development, holistic medicine, public health, Denmark

## Abstract

Despite the widespread acceptance of quality of life (QOL) as the ideal guideline in healthcare and clinical research, serious conceptual and methodological problems continue to plague this area. In an attempt to remedy this situation, we propose seven criteria that a quality-of-life concept must meet to provide a sound basis for investigation by questionnaire. The seven criteria or desiderata are: (1) an explicit definition of quality of life; (2) a coherent philosophy of human life from which the definition is derived; (3) a theory that operationalizes the philosophy by specifying unambiguous, nonoverlapping, and jointly exhaustive questionnaire items; (4) response alternatives that permit a fraction-scale interpretation; (5) technical checks of reproducibility; (6) meaningfulness to investigators, respondents, and users; and (7) an overall aesthetic appeal of the questionnaire. These criteria have guided the design of a validated 5-item generic, global quality-of-life questionnaire (QOL5), and a validated 317-item generic, global quality-of-life questionnaire (SEQOL), administered to a well-documented birth cohort of 7,400 Danes born in 1959—1961, as well as to a reference sample of 2,500 Danes. Presented in outline, the underlying integrative quality-of-life (IQOL) theory is a meta-theory. To illustrate the seven criteria at work, we show the extent to which they are satisfied by one of the eight component theories. Next, two sample results of our investigation are presented: satisfaction with one's sex life has the expected covariation with one's quality of life, and so does mother's smoking during pregnancy, albeit to a much smaller extent. It is concluded that the methodological framework presented has proved helpful in designing a questionnaire that is capable of yielding acceptably valid and reliable measurements of global and generic quality of life.

## INTRODUCTION

In recent years, quality of life (QOL) has become a key concept in the medical community[1,2,3,4,5,6,7,8], where healthcare places dual emphasis on the quality of care, in a technical sense, and client quality of life. Conjoining these two perspectives is a task for which the quality-of-life concept is especially well suited because it comprises both objective and subjective aspects: the personal, subjective experience of one's own life, as well as the more objective assessment of external factors that influence its quality.

Research designed to measure quality of life, however, suffers from various problems relating to theory and method, as several writers have pointed out[5,6,7,8]. Prominent is the poverty of theoretical foundation. Without an overarching theory of quality of life to guide the design of instruments, it is difficult to determine what to measure and how. Related to this is the problem of validation. Instruments are typically checked only by observation-to-observation cross-checks, and rarely by reference to theory, let alone to the experience of the respondents: Do they feel their quality of life is assessed correctly by the instrument?

We have carried out a quality-of-life questionnaire investigation of nearly 10,000 participants in a manner intended to overcome these problems. This paper presents the methodological framework employed, sketches a theory that integrates subjective and objective quality of life, and provides two sample illustrations of the results obtained.

## SEVEN CONCEPT CRITERIA

The methodology underlying this research is presented in terms of seven criteria or desiderata that must be met for a quality-of-life concept (by which we mean a generic quality-of-life concept as opposed to a more narrow, health-related, e.g., rheumatological, quality-of-life scale) to become a scientifically sound concept (see Table 1). Each criterion will be explained in more detail below.

**TABLE 1**
**Seven Criteria or Requirements for a Quality-of-Life Concept that can Provide a Sound Basis for Scientific Investigation by Questionnaire**

1. A definition of quality of life
2. An embedding philosophy of human life
3. A theory that operationalizes this philosophy by deriving questions that are unambiguous, nonoverlapping, and jointly exhaustive, and assigning relative weights to these questions
4. Quantifiable response alternatives
5. Technical checks (reproducibility, sensitivity, well scaledness, etc.)
6. Validation through meaningfulness to investigators, respondents, and users
7. Aesthetic appeal of the questionnaire

## A Definition of Quality of Life

For research, it is very important to have a common definition so that scientists can communicate and talk the same language. A clear definition also decreases the likelihood that healthcare policy makers and other users of the research results will misrepresent them.

## A Coherent Philosophy of Human Life

To have a philosophy from which the definition of quality of life is derived is imperative. We take it as axiomatic that any notion of quality of life is predicated on a particular view of life, and one ignores this at one's own risk. An atheoretical approach of the all-too-common kind may look less contrived, hence more objective, but is more likely to involve arbitrary elements, or even lifestyle-related prejudice and contradictory ideas.

## A Theory

The philosophy of life must be operationalized and made amenable to practical scientific investigation. A theory serves this function by structuring reality in such a way that particular aspects stand out and call for scrutiny. To this end, relevant questions are formulated. They must be unambiguous, nonoverlapping, and jointly exhaustive. The profusion of vague and repetitious questions that characterizes many purportedly global quality-of-life questionnaires is indicative of inadequate theoretical guidance. Poor theoretical preparation invariably returns to haunt the investigation after data collection, as all available office space fills with questionnaires that no one knows how to score.

A good theory also establishes the relative weights given to each question, that is, the influence each response has on the overall measure of quality of life. All too often, no serious theoretical justification is given for the weights assigned. Purely statistical weights, derived by regression or "factor" analysis, are not satisfactory, as the application of statistical procedures in itself does not guarantee meaningfulness.

## Quantifiable Response Alternatives

Each question must be given a set of response alternatives that can be interpreted quantitatively in accordance with the underlying theory, preferably as fractions (e.g., percentages) of the theoretically given span from minimum to maximum. For example, a patient's perceived mobility should not merely be expressed as "very good", "good", "fair", or "poor", as such categories are not easily scored. Higher-level measurement scale properties must be sought. A minimal requirement is an interval scale, allowing averages to be calculated and additive effects to be discussed. In our context, the theory adopted is likely to require that the scale has a bottom and a top, which allows intermediate positions to be interpreted on a fraction scale, that is, as fractions or percentages[9].

## Conventional Technical Checks

Checks of reproducibility (reliability), sensitivity (responsiveness, however conceived), and well-scaledness must be performed. These checks, however, should not stand alone, as they often appear to do in the literature. In fact, there is an unseemly tradition in health profile research according to which casually selected and interpreted correlation coefficients are uncritically adduced as evidence of validity and usefulness.

### Validity Through Meaningfulness

Participants in the research and those who use its results must find the elements of the research that they encounter meaningful. They must see the underlying theory, the questions asked (the issue of face validity), the responses given, and the analysis performed as transparent (no hidden agendas), intelligible (not overly complex), and reasonable. Specifically,

1. Investigators administering a questionnaire developed elsewhere must find the theory, the questions, the response alternatives, and the scaling meaningful.
2. The respondents must find each individual question meaningful and must feel that the questions collectively express their quality of life.
3. Those for whom the results are intended must be able to understand the results and the underlying theory.

These criteria aim to prevent the administration of questionnaires that the investigators do not fully understand and the respondents feel alienated by, and the results of which are subsequently misinterpreted by healthcare policy makers.

### The Aesthetic Dimension

Albeit a desiratum of a different kind, we hold it mandatory that the aesthetic dimension is given due attention. Not only the content, but also the composition of a questionnaire is important, as is a large number of other contextual factors: typography, layout, diagrams, sequence of questions, choice of words, the tone of the instructions. A questionnaire must feel right, and designing it is an art. Too many questionnaires in use appear disorganized, graphically unattractive, and uninspired. How can we expect subjects to reveal their quality of life if we show little regard for quality in our instruments?

## AN INTEGRATIVE QUALITY-OF-LIFE THEORY

Having presented these seven criteria, the central question is whether or not it is possible at all to construct a fair theory of quality of life. The problem grows if we intend to bridge the gap between the subjective and the objective sides of life, as these in many studies seem to be poorly correlated statistically. To solve the problem about how to measure the total quality of life for a person, we have to understand what constitutes the bridge, or bridges, between the subjective and the objective side of a person. That has been a hard philosophical question that many philosophers, like Kierkegaard, or researches like Frankl, Antonovsky, and Maslow to list a few, have tried to solve. It seems that, in between these two sides, we have an important third aspect of quality of life, which in existentialistic philosophy often is called the existential dimension. A quality-of-life and health theory based on this idea of an existential core is presented elsewhere[10].

A philosophical solution to the existential core perspective is to add deep quality-of-life dimensions like “the meaning of life” and “biological order” to the well-known dimensions of subjective and objective quality of life[11,19]. A simple five-question–questionnaire (QOL5) is successfully based on the IQOL theory[11].

Unfortunately, it turns out that such a pretty attempt to explain quality of life entails huge difficulties for the practical implementation of the theory into a more detailed questionnaire: to really measure the meaning of life, the respondent must understand such a question of meaning of life and acknowledge it as relevant. It seems very difficult these days, at least for Danes, to answer such a question.

Turning to the practical route of solving the problem of measuring the total quality of life that we chose to follow, it seemed that a number of dimensions could be found that combined the unstructured subjective side with the structure that characterizes the objective side. Fulfillment of needs (Maslow) is such a compelling example: we have some structure, our needs, and the experience of fulfillment. Another component theory looks at satisfaction at work, at home, and in leisure time, using three temporal domains. We have chosen two more theories that look at life as organized as concentric circles of proximity and distance, and evaluated the subjective quality of satisfaction respective of the subjective quality of all the relationships in the (somewhat arbitrary number: five) domains. The subjective factors — well being, satisfaction, and happiness — can be seen as progressively closer to the existential core, and more and more distant from mind, and life's surface and the subject's immediate experiential sphere. On the objective side we can measure ability to function by a person's possessions ("having-ness"): what do you have in your life? (Organized according to the five spatial domains: [1] money and health, [2] partner, [3] family and friends, [4] job status and social status, [5] environmental factors: nature and culture).

The IQOL theory that underlies our research is a meta-theory that integrates these several quality-of-life theories by organizing them along a spectrum from subjective to objective quality of life (see Table 2)[11,19]. Three of them concern subjective quality of life and are listed in increasing order of existential depth: quality of life as immediate, self-experienced well being[12] (No. 1 in Table 2), as satisfaction in life[13,14] (No. 2), and as happiness ad modum Aristotle (No. 3). The fourth concerns objective quality of life and lists a number of things that may or may not be obtained: does the respondent have a partner, children, a job, and hobbies?[15,16](No. 8).

**TABLE 2**
**The IQOL Theory**

Subjective

1. Immediate self-experienced well being
2. Satisfaction
3. Happiness
4. Fulfilment of Needs
5. Experience of objective temporal domains
6. Experience of objective spatial domains
7. Expression of Life's potential
8. Objective factors

Objective

The two remaining theories are about human nature: Maslow's theory of human needs[17] further elaborated by Aggernæs[12] (No. 4), and a theory of human beings seen as striving to express the potentials of life[17,18](No. 7). In their concern with the deeper levels of human existence, these two theories are taken to mediate between the subjective and objective sides of human being and are thus placed between these extremes.

The IQOL meta-theory allowed for two additional theories that blended the subjective and the objective ends of the spectrum by eliciting the respondents subjective experience of two sets of objective factors: the temporal (No. 5), respective spatial (No. 6 and 7) organization of their lives. ("Temporal organization" refers to the manner in which modern life was divided between work, family, and leisure, and "spatial organization" referred to the socio-spatial domains or levels presented under item 3 below.).

The quality of life assessment afforded by each of the eight constituent theories aspired to be global (covering all aspects of the life of a person, not just those related to a particular dysfunction) and generic (applicable to all persons, not just those belonging to a particular disease category). Organized under the IQOL umbrella, the instruments derived from the eight theories further attempted a comprehensive quality-of-life assessment that included a broad spectrum of subjective and objective factors.

A global, generic, and comprehensive measure of quality of life should be useful whenever a well-rounded description of the consequences of treatment or other intervention is called for. The eight constituent quality-of-life theories can be individually and collectively assessed for covariation with any variable of interest and thus provide both an aggregate quality-of-life measure and, when needed, a suitably detailed view.

## SATISFYING THE CONCEPT CRITERIA

For the IQOL meta-theory to be viable, each of the eight constituent theories must meet the seven criteria discussed above. We exemplify by showing how one of them — the theory of humans as beings expressing the potentials of life — addresses the criteria, this list doubling as a summary presentation of the theory. Similar analyses of the other theories are presented elsewhere[19].

1. Definition: Quality of life resides in the full expression of the potentials of life.
2. Philosophy: A human being is a biopsychosocial being who, through the expression of the potentials of life, strives to build a bridge between a deep (“inner”) world and the outer world at large.
3. Theory: Human life is hierarchically organized, with levels ranging from organic molecules through cells to the organism or the self, and further up through the dyadic relationship (partner), the close family (parents and children), friends and acquaintances (including siblings), and finally to the world at large (society and biosphere). While the first four levels are beyond control and direct subjective monitoring, the level of “self” is where subjective experience and control emerges. The self and its body is subjectively, as well as socially, acknowledged as the level of maximum control (there is less control over organs or cells, etc., and also less control over external things and beings), and hence it is also the level of maximum relevance in the context of realizing an inborn potential. Therefore only the last five levels, from self to world, are considered here. For life to express itself fully, there must be quantity and quality at each level in the hierarchy. A level must feature a certain number of elements (there must be a self, one must have a partner, a family, friends, and a larger social world) and they must be experienced as good. All levels are here considered of equal importance. This theory is an operationalization of the philosophy, in that the questions to be included in the questionnaire must ascertain the quantity and quality of each level; for example, "Do you have a mate?" and "How good is the relationship?" The questions are as unambiguous as one can expect in real life (a partner is a partner), nonoverlapping (a sibling is not a partner), and jointly exhaustive (quantity and quality of all five levels are included). Further, the relative weighting of the questions is given by the theory: Equal weights.

4. The qualitative questions in this theory are given quantifiable response alternatives by a scale construction that combines the core properties of a numerical scale, a visual analogue scale, and the Likert attitude scale. The resulting scale specifies a range from 0 to 100% of the theoretical maximum and has named gradations. Five symmetrically arranged response alternatives may be derived of the type "very bad", "bad", "neither good nor bad", "good", and "very good", interpretable as 10, 30, 50, 70, and 90% of the theoretical maximum. Thus, a fraction scale has been obtained.
5. Technical checks. Reliability: a 3-month test-retest yielded a correlation of $\underline{r}$ = 0.71 (within a subsample aged 33 of the reference sample mentioned below). Sensitivity: a between-groups difference of 3% requires 113 respondents in each “group” for detection, a 10% difference requires 12 respondents, and a 20% difference requires 4 respondents.
6. Meaningfulness to: (a) Investigators: we designed the questionnaire to our satisfaction; (b) Respondents: in response to the question "How well do these questions (representing the theory of quality of life as the expression of life's potentials) express your quality of life?", 77.6% of the reference sample answered "well" or "very well", and 2.8% answered "badly" or "very badly" (n = 1,436); (c) Users: as yet unascertained.
7. Aesthetic appeal: to our satisfaction. Readers of this journal can find the questionnaire as a part of the SEQOL questionnaire in Ventegodt[19] (English translation) for personal evaluation.

## RESPONDENTS

A well-documented cohort[20,21] comprising 7,400 children born at the University Hospital (Rigshospitalet), Copenhagen, in 1959–1961 presented a rare opportunity to correlate global quality of life in a sample of healthy subjects with various birth-related events. As a reference group, a representative, age-stratified population sample of 2,500 Danes aged 18–80 years was selected. A 317-item quality-of-life self-assessment questionnaire (SEQOL) including the 5-item quality-of-life self-assessment questionnaire (QOL5) were developed and administered to both groups. Both questionnaires have been validated[11,22].

## EMPIRICAL RESULTS

The response rate in this nonpatient study was 62% for the cohort and 61% for the reference sample. Mean answering time reported for the 317-item questionnaire was 49 min[23].

Two examples of the covariation of the eight quality-of-life measures with a variable of interest are given in Tables 3 and 4 and plotted in Figs. 1 and 2, respectively. Table 3 presents data from the reference sample on sexual satisfaction. In response to the question "How satisfied are you with your sex life as it is (or is not) at present?", respondents ticked one of the five alternatives listed in the leftmost column of the table. For each of these response categories, the mean quality-of-life score for each of the eight quality-of-life measures is given. Fig. 1 shows our preferred way of standardizing such results across the eight quality-of-life theories. Within each, the five quality-of-life means are expressed as deviations from their overall mean, and these deviations are rendered as percentages of that mean (the bona fide legitimacy of interpolating along the y-axis rests on the ordering of the eight quality-of-life theories being dictated by theory).

The resulting plot, which we call the IQOL landscape, shows an overall incline with increasing satisfaction with one's sex life, indicating that quality of life varies monotonely and, in fact, roughly linearly with such satisfaction. The fairly uniform incline of the landscape in all of the eight theories shows that this positive covariation applies to objective as well as subjective quality of life, although objective quality of life is less strongly associated with sexual satisfaction. This result agrees with expectation.

**TABLE 3**
**Degree of Satisfaction with Sex Life (Data from a Reference Sample of Danes, n = 1,397)**

| Degree of Satisfaction | (Score) | Mean of Quality-of-Life Measures (Range 0–100) | | | | | | | |
|---|---|---|---|---|---|---|---|---|---|
| | | 1 | 2 | 3 | 4 | 5 | 6 | 7 | 8 |
| Very satisfied | (0.9) | 79.5 | 77.8 | 75.8 | 75.8 | 82.1 | 75.3 | 51.2 | 75.1 |
| Satisfied | (0.7) | 74.0 | 71.9 | 67.3 | 71.0 | 77.0 | 70.7 | 47.4 | 72.4 |
| Neither/nor | (0.5) | 68.4 | 65.2 | 60.3 | 64.0 | 72.4 | 65.2 | 41.9 | 66.3 |
| Dissatisfied | (0.3) | 63.7 | 58.8 | 54.9 | 62.2 | 69.4 | 62.5 | 39.2 | 64.4 |
| Very dissatisfied | (0.1) | 54.5 | 48.8 | 48.8 | 57.7 | 65.4 | 57.7 | 36.3 | 62.9 |
| Overall mean | | 72.2 | 69.5 | 65.6 | 69.1 | 75.9 | 69.1 | 45.7 | 70.4 |

Note: For the different QOL measures 1-8, see Table 2.

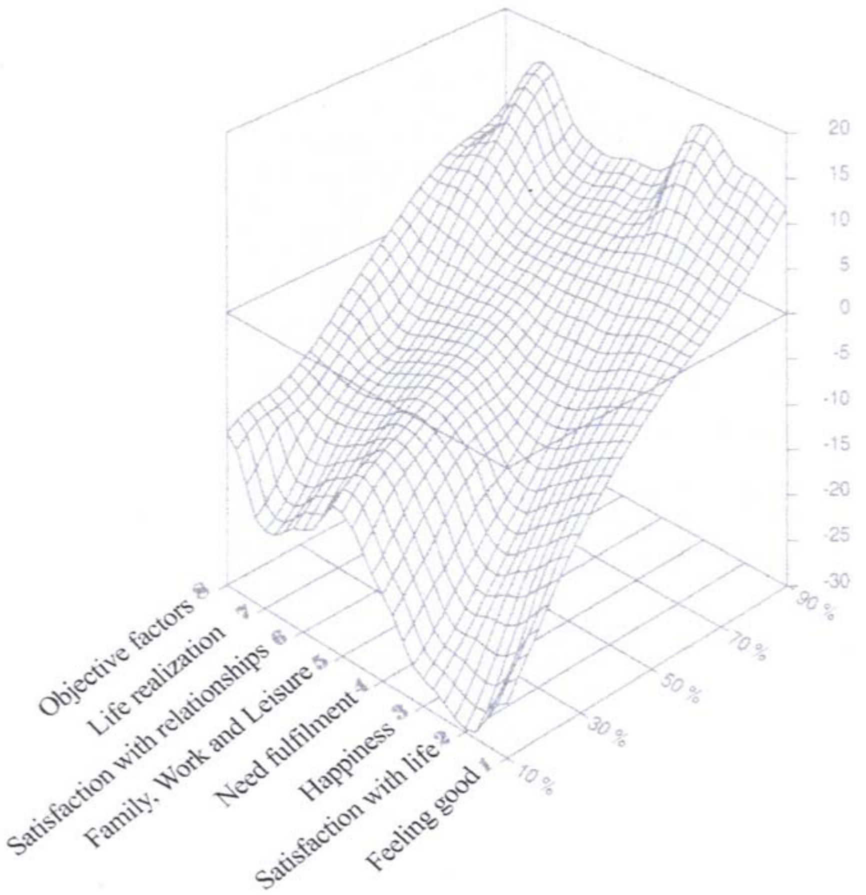

**FIGURE 1.** Quality of life (z-axis), as measured according to eight quality-of-life theories (y-axis), against satisfaction with sex life (x-axis). Data from Table 3, rescaled.

**TABLE 4**
**Maternal Smoking in the Third Trimester (Data from a Cohort of Danes Born between 1959–1961, n = 4,562)**

| Number of Cigarettes Smoked by Respondent's Mother During Her Last Trimester | Mean of Quality-of-Life Measure (Range 0–100) | | | | | | | |
|---|---|---|---|---|---|---|---|---|
| | 1 | 2 | 3 | 4 | 5 | 6 | 7 | 8 |
| <3 | 72.0 | 69.4 | 66.3 | 68.4 | 74.8 | 67.2 | 45.7 | 72.9 |
| 3–10 | 72.4 | 69.8 | 66.2 | 68.8 | 75.4 | 67.3 | 46.1 | 72.3 |
| >10 | 70.9 | 68.2 | 65.4 | 67.4 | 74.6 | 66.2 | 44.9 | 71.2 |
| Overall mean | 71.9 | 69.4 | 66.2 | 68.4 | 74.9 | 67.1 | 45.7 | 72.5 |

Note: For the different QOL measures 1-8, see Table 2.

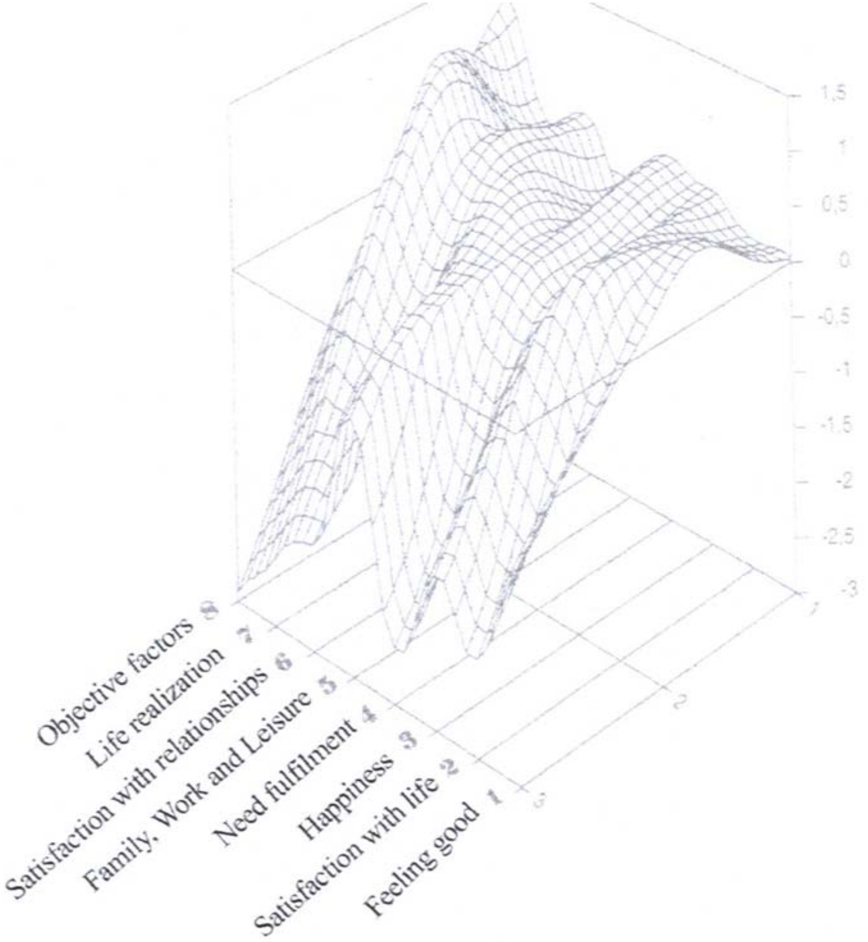

**FIGURE 2.** Quality of life (z-axis), as measured according to eight quality-of-life theories (y-axis), against maternal smoking in the third trimester (x-axis). Data from Table 4, rescaled.

Table 4 and Figure 2 show data from the cohort study on maternal smoking during the last trimester. The IQOL landscape shows that across the eight quality-of-life dimensions, heavy smoking by the mother during this period was fairly uniformly associated with a low quality-of-life score reported by the respondent some 30 years later. This pattern reinforces widespread suspicions about lingering ill effects of heavy maternal smoking and, to our knowledge, does so over a time span longer than previously reported. Although the association is not particularly strong, it seems substantial enough to warrant a search for causes.

## DISCUSSION

There is a burgeoning consensus in the healthcare community that the goal of modern medicine is not merely the prolongation of life, but its betterment. We believe the time is ripe to expand the scope of quality-of-life research from the current, somewhat narrow focus on treatment evaluation to more general prevention efforts that aim to monitor and help improve the health status of the population at large. Quality-of-life factors that escape notice in the commonplace questioning about occupational and functional disabilities may well be crucial in precipitating manifest illness. The value of the concept of quality of life lies in its use, not solely for the purpose of evaluating therapy, but also for guiding preventive research and goal setting. We therefore need instruments that fully characterize and measure the quality of life of patients and citizens.

We have attempted to contribute to the development of theory and methodology in the quality-of-life field by suggesting seven fairly demanding criteria for quality-of-life research by questionnaire. These criteria influenced the design of an extensive questionnaire that was administered to two large samples of participants. The two results presented demonstrate the viability of the instrument.

The underlying IQOL theory is a meta-theory with eight constituent theories spanning the spectrum of human existence from subjective to objective factors. The results presented here and elsewhere suggest that it is possible to obtain an assessment of quality of life that is global, generic, and comprehensive.

Comprehensive as the IQOL measures are, we do not believe we have succeeded in tapping the deepest aspects of human experience, those relating to purpose in life, and the farther reaches of the human spirit. Further developments will no doubt acknowledge the depths of human’s existence to a higher extent. Still, spiritual aspects may well be beyond verbal articulation. They are, however, crucial in establishing the priorities in life for the individual person. For this reason, it is important to remember that verbal quality-of-life surveys like the one discussed here can be no more than rough guides to decision makers and should be no substitute for soliciting the opinion of the individual person and groups of persons of a proposed treatment or other course of action.

## ACKNOWLEDGMENTS

This study was supported by grants from The 1991 Pharmacy Foundation, as well as by supplementary grants from Goodwill-fonden, the JL-Foundation, E. Danielsen and Wife's Foundation, Emmerick Meyer's Trust, the Frimodt-Heineken Foundation, the Hede Nielsen Family Foundation, Petrus Andersens Fond, Wholesaler C.P. Frederiksens Study Trust, Else & Mogens Wedell-Wedellsborg's Foundation, and IMK Almene Fond. We gratefully acknowledge the critical scrutiny and expert linguistic assistance of Ib Ravn, Ph.D. The research was approved by the Copenhagen Scientific Ethical Committee under number (KF)V.100.2123/91. This paper was possible due to efforts of the quality of life study group at the University of Copenhagen[24].

## BIOSKETCHES

**Søren Ventegodt, MD,** is the Director of the Quality of Life Research Center in Copenhagen, Denmark. He is also responsible for a Research Clinic for Holistic Medicine in Copenhagen and is a popular speaker throughout Scandinavia. He has published numerous scientific or popular articles and a number of books on holistic medicine, quality of life, and quality of working life. His most important scientific contributions are the comprehensive SEQOL questionnaire, the very short QOL5 questionnaire, the integrated quality-of-life (IQOL) theory, the holistic process theory, the life mission theory, and the Danish Quality of Life Research Survey, 1991–94 in cooperation with the University Hospital of Copenhagen and the late pediatric professor Bengt Zachau-Christiansen. E-mail: ventegodt@livskvalitet.org. Website: www.livskvalitet.org/

**Jørgen Hilden, PhD,** is Professor in the Department of Biostatistics, Institute of Public Health, Faculty of Health Sciences, University of Copenhagen, Denmark. His research interests are statistical methods in medical diagnosis, decision analysis, rational clinical management, and meta-analysis. E-mail: J.Hilden@biostat.ku.dk. Website: www.biostat.ku.dk/staff/jh-e.htm#research

**Joav Merrick, MD, DMSc,** is Professor of Child Health and Human Development affiliated with the Zusman Child Development Center and Division of Community Health at the Ben Gurion University, Beer-Sheva, Israel and presently the Medical Director of the Division for Mental Retardation, Ministry of Social Affairs, Jerusalem and the Director of the National Institute of Child Health and Human Development. He has numerous publications in the field of child and human development, rehabilitation, intellectual disability, disability, health, welfare, abuse, advocacy and prevention. Dr. Merrick received the Peter Sabroe Child Award for outstanding work on behalf of Danish Children in 1985 and the International LEGO-Prize ("The Children's Nobel Prize") for an extraordinary contribution towards improvement in child welfare and well being in 1987. E-mail: jmerrick@internet-zahav.net. Website: www.nichd-israel.com
